# HS‐10352 in hormone receptor‐positive, HER2‐negative advanced breast cancer: A phase 1 dose‐escalation trial

**DOI:** 10.1002/cam4.6755

**Published:** 2023-12-01

**Authors:** Quchang Ouyang, Ying Wang, Jian Zhang, Qiong Wu, Hongying Wei, Chuan Li, Xiaoling Qian, Xichun Hu

**Affiliations:** ^1^ Department of Breast Medicine Hunan Cancer Center Changsha Hunan China; ^2^ Department of Breast Oncology, Sun Yat‐Sen Memorial Hospital Sun Yat‐Sen University Second Affiliated Hospital of Sun Yat‐Sen University Guangzhou China; ^3^ Department of Medical Oncology Fudan Shanghai University Cancer Center Shanghai China; ^4^ Clinical Research Center, Shanghai Hansoh Biomedical Co., Ltd. Shanghai China

**Keywords:** breast cancer, dose escalation, hormone receptor, HS‐10352, human epidermal growth factor receptor 2

## Abstract

**Background:**

Approximately 40% of patients with hormone receptor (HR)‐positive and human epidermal growth factor receptor 2 (HER2)‐negative advanced breast cancer (ABC) exhibit PIK3CA mutations.

**Aims:**

This study aims to evaluate the safety, tolerability, pharmacokinetics, and preliminary antitumor activity of HS‐10352, a selective PI3Kα inhibitor, in this patient population.

**Materials and Methods:**

Conducted as a phase 1 dose‐escalation trial, HS‐10352 was administered orally once‐daily (QD) at dose levels of 2, 4, 6, and 8 mg. The primary endpoints were dose‐limiting toxicity (DLT) and the maximum tolerated dose (MTD). This study is registered at ClinicalTrials.gov (NCT04631835).

**Results:**

Between August 2020 and March 2022, a total of 18 female patients were enrolled. DLT, manifested as hyperglycemia, occurred in two patients in the 8 mg QD group, establishing an MTD of 6 mg QD. The most common treatment‐related adverse events were hyperglycemia (88.9%) and weight loss (61.3%). In the 6 mg QD group, four patients (66.7%) had a partial response (PR), and one (16.7%) had stable disease (SD). Among the four patients with PIK3CA mutated tumors in this dosage group, three (75.0%) had PR and one (25.0%) had SD. The median progression‐free survival was not reached (95% confidence interval, 11.1‐NA).

**Discussion and Conclusion:**

HS‐10352 at 6 mg QD was well‐tolerated in patients with HR‐positive, HER2‐negative ABC, and showed preliminary antitumor activity in patients with PIK3CA mutated tumors. These findings support the further clinical development of HS‐10352.

## Graphical abstract

HS‐10352 is a novel selective PI3Kα inhibitor showing inhibitory effects on the proliferation of cancer tumor cell lines harboring PIK3CA mutations. This study first evaluated the safety, tolerability, pharmacokinetics, and preliminary antitumor activity of HS‐10352 in patients with HR‐positive, HER2‐negative metastatic breast cancer. The results support further clinical development of HS‐10352.

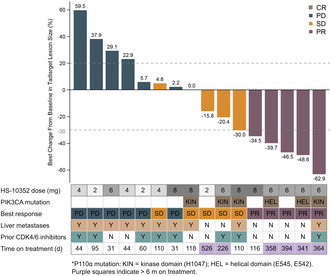

## 1 INTRODUCTION

Breast cancer is the most common cancer and the leading cause of cancer‐related deaths in females.^1^ Hormone receptor (HR)‐positive, human epidermal growth factor receptor 2 (HER2)‐negative subtype accounts for over two‐thirds of all breast cancer cases.^2^, ^3^ The current standard of care for HR‐positive, HER2‐negative advanced breast cancer (ABC) involves endocrine therapy, with or without cyclin‐dependent kinase 4/6 (CDK4/6) inhibitors.^4^, ^5^, ^6^ PIK3CA mutations are present in approximately 40% of patients with this subtype,^3^, ^7^ with a higher prevalence in the Chinese population than Whites.^8^ The SOLAR‐1 trial demonstrated that adding the PI3Kα inhibitor alpelisib to fulvestrant significantly improved progression‐free survival (PFS) in patients with PIK3CA‐mutated, HR‐positive, HER2‐negative ABC who had received prior endocrine therapy.^9^ The median PFS after alpelisib and fulvestrant treatment in the SOLAR‐1 trial was 11.0 months, superior to 5.7 months in the placebo plus fulvestrant group.^9^ However, evidence demonstrating the efficacy of PI3Kα inhibitors in Chinese patients with ABC is scarce.

HS‐10352 is a novel, highly selective PI3Kα inhibitor with an IC50 value of 0.34 nM. It exhibits far greater selectivity for PI3Kα over PI3Kβ, PI3Kδ, and PI3Kγ, with 321‐, 103‐, and 159‐fold higher selectivity, respectively. This heightened selectivity may help minimize the severe side effects commonly associated with the inhibition of PI3Kβ, δ, and γ. Preclinical studies, both in vitro and in vivo, have demonstrated that HS‐10352 effectively inhibits PIK3CA mutations. For instance, HS‐10352 substantially inhibited the proliferation of human breast cancer cell lines harboring PIK3CA mutations, such as HCC1954 and MCF‐7, in vitro assays. Conversely, HS‐10352 displayed no inhibitory effects on cell lines lacking PIK3CA mutations, like the human breast cancer HDQ‐P1 cell line and the human gastric cancer SNU‐1 and MKN‐74 cell lines. In animal models, HS‐10352 markedly suppressed the growth of transplanted tumors in a dose‐dependent manner in mice bearing gastric cancer HGC‐27 (PIK3CA E542K) and human breast cancer HCC‐1954 (PIK3CA H1047R) tumors. Specifically, in mice with HCC‐1954 tumors, oral treatment with HS‐10352 at 10 mg/kg once‐daily (QD) for 21 days resulted in a 124.47% inhibition in tumor growth without causing significant weight loss.

This study aimed to evaluate the safety, tolerability, pharmacokinetics, and preliminary antitumor efficacy of oral HS‐10352 in patients with HR‐positive, HER2‐negative ABC.

## 2 METHODS

### 2.1 Study design and patients

This was an open‐label, phase 1 dose‐escalation trial registered at ClinicalTrials.gov (NCT04631835). This trial was conducted at three medical centers in China from August 2020 to March 2022 (Table S1). The study protocol was approved by the ethics committee of Fudan University Shanghai Cancer Center (No. 2006218–20), and all participants provided written informed consent prior to enrolment.

Key inclusion criteria were as follows: (1) 18–75 years of age; (2) at least one measurable lesion according to Response Evaluation Criteria In Solid Tumors (RECIST) version 1.1^10^; (3) histologically or cytologically diagnosed HR‐positive, HER2‐negative ABC; (4) patients with tumors that did not respond to the standard of care, for whom standard treatments were unavailable, or unable to tolerate the toxicities of the standard therapies; (5) Eastern Cooperative Oncology Group (ECOG) performance status of 0–1; and (6) expected life expectancy ≥3 months; and (7) adequate organ functions.

Key exclusion criteria were as follows: (1) previous treatment with PI3K, AKT, or mTOR inhibitors before enrollment; (2) previous treatment with potent CYP3A4 and/or CYP2C8 inhibitors or inducers within 7 days before enrollment; (3) unstable, untreated or newly onset brain metastases, spinal cord compression, or meningeal/brain stem metastases; (4) history of other primary malignant tumors; (5) diabetes symptoms or a history of diabetes; (6) acute or chronic pancreatitis; or (7) other severe diseases that might affect the safety and efficacy of patients.

### 2.2 Procedure and assessments

This dose‐escalation trial employed a “Rolling 6” design,^11^, ^12^ accommodating a maximum of six patients in each dose group. HS‐10352 was administered orally at varying dose levels (2, 4, 6, and 8 mg) QD. If a dose‐exposure plateau or the maximum tolerated dose (MTD) was not reached by the end of this study, consideration for further dose escalation would be discussed by the investigators and the sponsor, based on the pharmacokinetic and safety profiles of the different dose groups.

Blood samples were collected from each patient after a single dose of HS‐10352 on Day 1 of Cycle 0, followed by a 7‐day observation period. Repeated administration of the same dose commenced on Day 8, marking the beginning of Cycle 1. Each treatment cycle lasted for 4 weeks. Treatment with HS‐10352 would be discontinued in case of disease progression, adverse events (AEs), serious protocol violations, withdrawal of consent, or other circumstances as determined by the investigators or the sponsor.

Treatment response was assessed by the investigators every 8 weeks according to RECIST v1.1. Patients were followed up for safety for 28 days after treatment discontinuation. Any AEs occurring during the trial were recorded and graded according to the National Cancer Institute Common Terminology Criteria for Adverse Events (CTCAE) version 5.0,^13^ summarizing the incidence rates of AEs, treatment‐related AEs (TRAEs), serious AEs (SAEs), and grade ≥3 AEs. The proportions of patients who underwent dose adjustment or discontinuation due to AEs were also reported.

### 2.3 PIK3CA mutation test

Breast tumor tissue samples were collected, and PIK3CA mutation status was determined by the central laboratory using the fluorescence quantitative PCR instrument (Cobas Z480) along with the PIK3CA Mutation Detection Kit (AmoyDx, Xiamen, China). This kit is specifically designed to detect the five hotspot PIK3CA mutations: H1047L, H1047R, E542K, E545K, and E545D.

### 2.4 Study endpoints

The primary endpoints were dose‐limiting toxicity (DLT) and MTD of HS‐10352. Secondary endpoints included safety, pharmacokinetics, objective response rate (ORR), disease control rate (DCR), duration of response (DoR), PFS, and overall survival (OS).

DLT was defined as any grade ≥4 hematologic or grade ≥3 non‐hematologic toxicity that occurred from the single dose on Day 1 of Cycle 0 up to the last dose in the first repeated dosing cycle, which was unrelated to the disease but associated with the study drug per investigator judgment according to CTCAE version 5.0. MTD was defined as either the highest tolerable dose at which DLT occurred in no more than one patient or as the intermediate dose between the intolerable dose (where DLT occurred in two or more patients) and the previously determined highest tolerable dose. At the intermediate dose level, DLT should occur in no more than one patient during subsequent assessments. ORR was defined as the proportion of patients with a confirmed complete response (CR) or partial response (PR). DCR was defined as the proportion of patients with a confirmed CR, PR, or stable disease (SD).

### 2.5 Statistical analysis

A “Rolling 6” design was adopted in this dose‐escalation study, with up to six evaluable patients enrolled in each dose group. The total number of participants was contingent on the number of dose‐escalation groups.

Statistical analyses primarily utilized descriptive statistics. Continuous variables were presented as mean ± standard deviation or median (range) depending on the normality of the data. Categorical variables were presented as frequency (percentage). The 95% confidence intervals (CIs) for the ORR and DCR were calculated using the Clopper–Pearson method. The Kaplan–Meier method was employed to estimate the medians and 95% CIs of PFS and DoR. Statistical analyses were conducted using SAS version 9.4 (SAS Institute, Cary, NC, USA).

## 3 RESULTS

### 3.1 Patient characteristics

**FIGURE 1:**
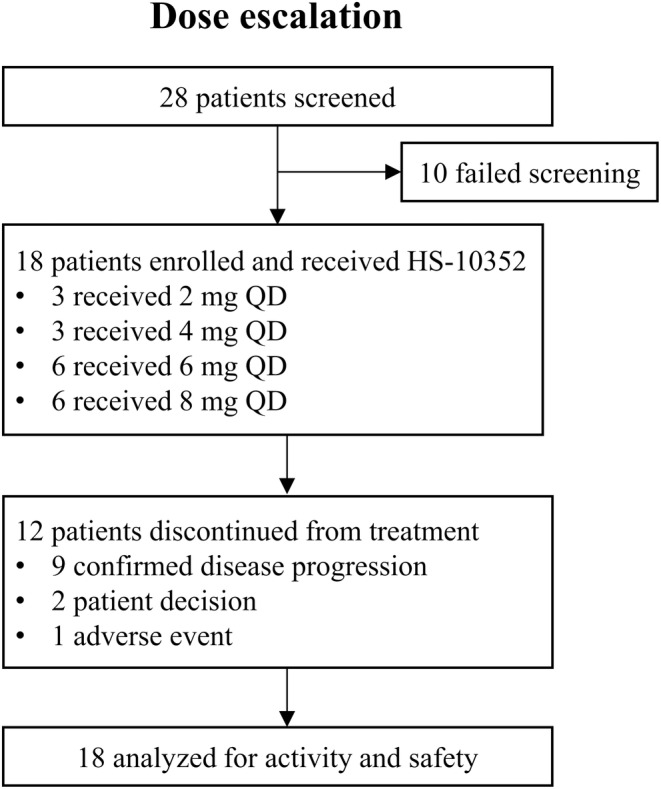
Study flowchart.

A total of 28 patients were screened for eligibility between August 2020 and March 2022. Of these, 18 female patients with HR‐positive, HER2‐negative ABC were enrolled and treated with HS‐10352 (Figure 1). The average age of the participants was 50.8 ± 8.50 years, and 12 (66.7%) cases had an ECOG performance status of 1. Central laboratory testing confirmed 6 (33.3%) patients with PIK3CA mutated tumors, 10 (55.6%) with PIK3CA wild‐type tumors, and two could not be assessed due to technical reasons. Furthermore, nine (50.0%) patients were confirmed to have PIK3CA mutations either by the central laboratory or based on their past medical records. All enrolled patients had visceral metastases: 11 (61.1%) had liver involvement, 1 (5.6%) had brain metastases, and 13 (72.2%) had bone metastases. The majority of patients had undergone a diverse range of treatments, including surgery (94.4%), endocrine therapy (94.4%), chemotherapy (94.4%), and targeted therapy (72.2%) prior to enrollment in this study. In terms of endocrine therapy, fulvestrant (12 [66.7%]) was the most commonly administered treatment, followed by letrozole (9 [50.0%]), anastrozole (7 [38.9%]), toremifene (7 [38.9%]), tamoxifen (6 [33.3%]), and exemestane (6 [33.3%]). Eight (44.4%) patients had prior exposure to CDK4/6 inhibitors, among which palbociclib (5 [27.8%]) was the most frequently used, followed by abemaciclib (2 [11.1%]), BEBT‐209 (1 [5.6%]), and unknown medication (1 [5.6%]). In addition, 4 (22.2%) patients had previously received anti‐angiogenic therapy, 3 (16.7%) had received histone deacetylase (HDAC) inhibitors, and 1 (5.6%) had received unknown targeted therapy (Table 1). The median number of prior systemic therapies for metastatic cancer was 4.

**TABLE 1:** Baseline patient characteristics.

Characteristic	2 mg QD	4 mg QD	6 mg QD	8 mg QD	Total
	(*N* = 3)	(*N* = 3)	(*N* = 6)	(*N* = 6)	(*N* = 18)
Age, years, mean ± SD	53.0 ± 4.58	56.0 ± 7.21	46.3 ± 7.97	51.5 ± 10.46	50.8 ± 8.50
Sex (female), *n* (%)	3 (100)	3 (100)	6 (100)	6 (100)	18 (100)
BMI, kg/m^2^, mean ± SD	24.80 ± 2.433	26.80 ± 5.381	22.47 ± 2.033	23.33 ± 2.252	23.87 ± 3.046
Smoking, *n* (%)					
Yes	0	0	0	0	0
No	3 (100)	3 (100)	6 (100)	6 (100)	18 (100)
ECOG PS, *n* (%)					
0	1 (33.3)	1 (33.3)	2 (33.3)	2 (33.3)	6 (33.3)
1	2 (66.7)	2 (66.7)	4 (66.7)	4 (66.7)	12 (66.7)
ER status, *n* (%)					
Positive	3 (100)	3 (100)	5 (83.3)	6 (100)	17 (94.4)
Negative	0	0	1 (16.7)	0	1 (5.6)
PR status, *n* (%)					
Positive	3 (100)	3 (100)	6 (100)	2 (33.3)	14 (77.8)
Negative	0	0	0	4 (66.7)	4 (22.2)
HER2 status, *n* (%)					
Positive	0	0	0	0	0
Negative	3 (100.0)	3 (100)	6 (100.0)	6 (100.0)	18 (100.0)
PIK3CA mutation^a^, *n* (%)					
Positive	0	0	4 (66.7)	2 (33.3)	6 (33.3)
Negative	3 (100)	2 (66.7)	2 (33.3)	4 (66.7)	11 (61.1)
Missing	0	1 (33.3)	0	0	1 (5.6)
PIK3CA mutation^b^, *n* (%)					
Positive	1 (33.3)	0	4 (66.7)	4 (66.7)	9 (50.0)
Negative	2 (66.7)	2 (66.7)	2 (33.3)	2 (33.3)	8 (44.4)
Missing	0	1 (33.3)	0	0	1 (5.6)
Metastatic sites, *n* (%)					
Bone	2 (66.7)	2 (66.7)	4 (66.7)	5 (83.3)	13 (72.2)
Brain	1 (33.3)	0	0	0	1 (5.6)
Liver	2 (66.7)	3 (100)	2 (33.3)	4 (66.7)	11 (61.1)
Other viscera	3 (100)	3 (100)	6 (100)	6 (100)	18 (100)
Prior antitumor therapy, *n* (%)					
Radiotherapy	2 (66.7)	2 (66.7)	3 (50.0)	2 (33.3)	9 (50.0)
Surgery	3 (100)	3 (100)	6 (100)	5 (83.3)	17 (94.4)
Local treatment	0	0	0	1 (16.7)	1 (5.6)
Endocrine therapy	3 (100)	3 (100)	6 (100)	5 (83.3)	17 (94.4)
Fulvestrant	3 (100)	3 (100)	4 (66.7)	2 (33.3)	12 (66.7)
Letrozole	2 (66.7)	1 (33.3)	3 (50.0)	3 (50.0)	9 (50.0)
Anastrozole	2 (66.7)	2 (66.7)	2 (33.3)	1 (16.7)	7 (38.9)
Toremifene	1 (33.3)	1 (33.3)	4 (66.7)	1 (16.7)	7 (38.9)
Tamoxifen	1 (33.3)	0	2 (33.3)	3 (50.0)	6 (33.3)
Exemestane	1 (33.3)	0	2 (33.3)	3 (50.0)	6 (33.3)
Targeted therapy	2 (66.7)	2 (66.7)	5 (83.3)	4 (66.7)	13 (72.2)
CDK4/6 inhibitor	2 (66.7)	2 (66.7)	2 (33.3)	2 (33.3)	8 (44.4)
Palbociclib	0	1 (33.3)	2 (33.3)	2 (33.3)	5 (27.8)
Abemaciclib	2 (66.7)	0	0	0	2 (11.1)
Others^c^	1 (33.3)	1 (33.3)	0	0	2 (11.1)
Anti‐angiogenic therapy	0	1 (33.3)	1 (16.7)	2 (33.3)	4 (22.2)
Bevacizumab	0	0	1 (16.7)	2 (33.3)	3 (16.7)
Anlotinib	0	1 (33.3)	0	0	1 (5.6)
HDAC inhibitor	1 (33.3)	0	1 (16.7)	1 (16.7)	3 (16.7)
Chidamide	1 (33.3)	0	1 (16.7)	0	2 (11.1)
Entinostat	0	0	0	1 (16.7)	1 (5.6)
Unknown	0	0	1 (16.7)	0	1 (5.6)
Chemotherapy	3 (100)	3 (100)	6 (100)	5 (83.3)	17 (94.4)
Immunotherapy	0	1 (33.3)	0	0	2 (11.1)
Others	1 (33.3)	1 (33.3)	1 (16.7)	1 (16.7)	4 (22.2)

Abbreviations: BMI, body mass index; CDK, cyclin‐dependent kinase; ECOG PS, Eastern Cooperative Oncology Group performance status; ER, estrogen receptor; HDAC, histone deacetylase; HER2, human epidermal growth factor receptor 2; PR, progesterone receptor; SD, standard deviation.

^a^ PIK3CA mutation confirmed by the central laboratory.

^b^ PIK3CA mutation confirmed by the central laboratory or past medical records.

^c^ Others included one case of BEBT‐209, a novel CDK4/6 inhibitor being investigated in clinical trials, and one case of unknown medication.

### 3.2 Safety

In the 8 mg QD group, two patients experienced DLTs of grade 3 hyperglycemia. No DLTs were reported in other dose groups, indicating an MTD of 6 mg QD. The incidence rates of AEs and grade ≥3 AEs were 100% and 27.8%, respectively. TRAEs occurred in all patients, regardless of the drug dose (Table S2). The most common TRAEs (≥20%) were hyperglycemia (16 [88.9%]), weight loss (11 [61.3%]), elevated insulin C‐peptide (6 [33.3%]), diarrhea (6 [33.3%]), elevated alanine aminotransferase (5 [27.8%]), elevated aspartate aminotransferase (5 [27.8%]), elevated blood insulin (5 [27.8%]), hypokalemia (5 [27.8%]), decreased appetite (5 [27.8%]), hypocalcemia (5 [27.8%]), nausea (5 [27.8%]), vomiting (5 [27.8%]), hypoalbuminemia (4 [22.2%]), hyponatremia (4 [22.2%]), sinus bradycardia (4 [22.2%]), urinary tract infection (4 [22.2%]), rash (4 [22.2%]), and proteinuria (4 [22.2%]) (Table 2). Hyperglycemic events were mainly grade ≤2, including 3 grade 1, 11 grade 2, and 2 grade 3 cases. Out of the 16 patients experiencing hyperglycemia, three did not require hypoglycemic therapy, two temporarily used insulin (both in the 8 mg QD group), and 11 were treated with oral hypoglycemic agents, achieving subsequent glucose control. Grade ≥3 TRAEs only occurred in the 6 mg QD group (weight loss [*n* = 2]) and the 8 mg QD group (hyperglycemia [*n* = 2], fatigue [*n* = 1], blurred vision [*n* = 1], hyperkalemia [*n* = 1] and hypocalcemia [*n* = 1]), with incidence rates of 33.3% (2/6) and 50.0% (3/6), respectively. One patient in the 6 mg QD group and two patients in the 8 mg QD group experienced dose reduction due to TRAEs. One patient in the 6 mg QD group had dose reduction due to grade 1 limb edema, grade 1 facial rash, and grade 2 rash. Two patients in the 8 mg QD group experienced dose reductions due to hyperglycemia. Only one patient (4 mg QD group) interrupted treatment due to a TRAE (hyperglycemia), and another (8 mg QD group) discontinued treatment due to grade 3 treatment‐related SAEs, including hyperglycemia, hyperkalemia, and elevated liver function biomarkers. No treatment‐related death occurred.

**TABLE 2:** TRAEs occurring in ≥10% of patients.

Event, *n* (%)	2 mg QD (*N* = 3)		4 mg QD (*N* = 3)		6 mg QD (*N* = 6)		8 mg QD (*N* = 6)		Total (*N* = 18)	
	Any grade	Grade 3–4	Any grade	Grade 3–4	Any grade	Grade 3–4	Any grade	Grade 3–4	Any grade	Grade 3–4
Hyperglycemia	3 (100.0)	0	2 (66.7)	0	6 (100.0)	0	5 (83.3)	2 (33.3)	16 (88.9)	2 (11.1)
Weight loss	2 (66.7)	0	1 (33.3)	0	6 (100.0)	2 (33.3)	2 (33.3)	0	11 (61.1)	2 (11.1)
Insulin C‐peptide elevation	0	0	1 (33.3)	0	3 (50.0)	0	2 (33.3)	0	6 (33.3)	0
Diarrhea	1 (33.3)	0	0	0	4 (66.7)	0	1 (16.7)	0	6 (33.3)	0
Alanine aminotransferase elevation	0	0	0	0	3 (50.0)	0	2 (33.3)	0	5 (27.8)	0
Aspartate aminotransferase elevation	1 (33.3)	0	1 (33.3)	0	2 (33.3)	0	1 (16.7)	0	5 (27.8)	0
Blood insulin elevation	0	0	1 (33.3)	0	2 (33.3)	0	2 (33.3)	0	5 (27.8)	0
Hypokalemia	2 (66.7)	0	1 (33.3)	0	1 (16.7)	0	1 (16.7)	0	5 (27.8)	0
Decreased appetite	0	0	0	0	3 (50.0)	0	2 (33.3)	0	5 (27.8)	0
Hypocalcemia	1 (33.3)	0	0	0	1 (16.7)	0	3 (50.0)	1 (16.7)	5 (27.8)	1 (5.6)
Nausea	0	0	0	0	2 (33.3)	0	3 (50.0)	0	5 (27.8)	0
Vomiting	0	0	0	0	3 (50.0)	0	2 (33.3)	0	5 (27.8)	0
Hypoalbuminemia	1 (33.3)	0	1 (33.3)	0	1 (16.7)	0	1 (16.7)	0	4 (22.2)	0
Hyponatremia	0	0	0	0	4 (66.7)	0	0	0	4 (22.2)	0
Sinus bradycardia	0	0	0	0	2 (33.3)	0	2 (33.3)	0	4 (22.2)	0
Urinary tract infection	1 (33.3)	0	1 (33.3)	0	1 (16.7)	0	1 (16.7)	0	4 (22.2)	0
Rash	1 (33.3)	0	0	0	3 (50.0)	0	0	0	4 (22.2)	0
Proteinuria	0	0	0	0	2 (33.3)	0	2 (33.3)	0	4 (22.2)	0
Bilirubin conjugated elevation	0	0	0	0	2 (33.3)	0	1 (16.7)	0	3 (16.7)	0
Blood creatinine elevation	0	0	0	0	1 (16.7)	0	2 (33.3)	0	3 (16.7)	0
QT prolongation on the electrocardiogram	1 (33.3)	0	0	0	2 (33.3)	0	0	0	3 (16.7)	0
Glycosylated hemoglobin elevation	0	0	1 (33.3)	0	1 (16.7)	0	1 (16.7)	0	3 (16.7)	0
Lymphocyte count decrease	1 (33.3)	0	0	0	1 (16.7)	0	1 (16.7)	0	3 (16.7)	0
Asthenia	0	0	0	0	2 (33.3)	0	1 (16.7)	0	3 (16.7)	0
Edema peripheral	0	0	0	0	2 (33.3)	0	1 (16.7)	0	3 (16.7)	0
Sinus arrhythmia	0	0	1 (33.3)	0	1 (16.7)	0	1 (16.7)	0	3 (16.7)	0
Anemia	1 (33.3)	0	0	0	1 (16.7)	0	1 (16.7)	0	3 (16.7)	0
Vision blurred	0	0	0	0	1 (16.7)	0	2 (33.3)	1 (16.7)	3 (16.7)	1 (5.6)
Headache	0	0	0	0	2 (33.3)	0	1 (16.7)	0	3 (16.7)	0
Blood lactate dehydrogenase elevation	0	0	1 (33.3)	0	1 (16.7)	0	0	0	2 (11.1)	0
Neutrophil count decrease	0	0	1 (33.3)	0	0	0	1 (16.7)	0	2 (11.1)	0
Protein in urine	0	0	1 (33.3)	0	1 (16.7)	0	0	0	2 (11.1)	0
White blood cell count decrease	0	0	0	0	1 (16.7)	0	1 (16.7)	0	2 (11.1)	0
Blood bilirubin elevation	0	0	0	0	1 (16.7)	0	1 (16.7)	0	2 (11.1)	0
Fibrin D‐dimer elevation	1 (33.3)	0	0	0	0	0	1 (16.7)	0	2 (11.1)	0
Glycated albumin elevation	0	0	1 (33.3)	0	1 (16.7)	0	0	0	2 (11.1)	0
Urinary occult blood positivity	0	0	1 (33.3)	0	1 (16.7)	0	0	0	2 (11.1)	0
Hyperlipidemia	0	0	1 (33.3)	0	1 (16.7)	0	0	0	2 (11.1)	0
Hypertriglyceridemia	0	0	0	0	1 (16.7)	0	1 (16.7)	0	2 (11.1)	0
Hypochloremia	0	0	0	0	2 (33.3)	0	0	0	2 (11.1)	0
Hypomagnesemia	0	0	0	0	2 (33.3)	0	0	0	2 (11.1)	0
Toothache	0	0	1 (33.3)	0	0	0	1 (16.7)	0	2 (11.1)	0
Abdominal pain	0	0	0	0	2 (33.3)	0	0	0	2 (11.1)	0
Abdominal pain upper	0	0	0	0	1 (16.7)	0	1 (16.7)	0	2 (11.1)	0
Flatulence	0	0	0	0	1 (16.7)	0	1 (16.7)	0	2 (11.1)	0
Face edema	0	0	0	0	1 (16.7)	0	1 (16.7)	0	2 (11.1)	0
Fatigue	0	0	0	0	1 (16.7)	0	1 (16.7)	1 (16.7)	2 (11.1)	1 (5.6)
Upper respiratory tract infection	0	0	1 (33.3)	0	1 (16.7)	0	0	0	2 (11.1)	0
Dizziness	0	0	0	0	2 (33.3)	0	0	0	2 (11.1)	0

The median treatment exposure for HS‐10352 was 110.5 days (range, 12 to 525). The exposure duration was the longest in the 6 mg QD group (288.8 ± 140.73 days), which also had the highest cumulative exposure dose (1632.0 ± 793.67 mg) (Table S3). The swimmer plot for the duration of treatment in individuals is shown in Figure S1.

### 3.3 Pharmacokinetics

**FIGURE 2:**
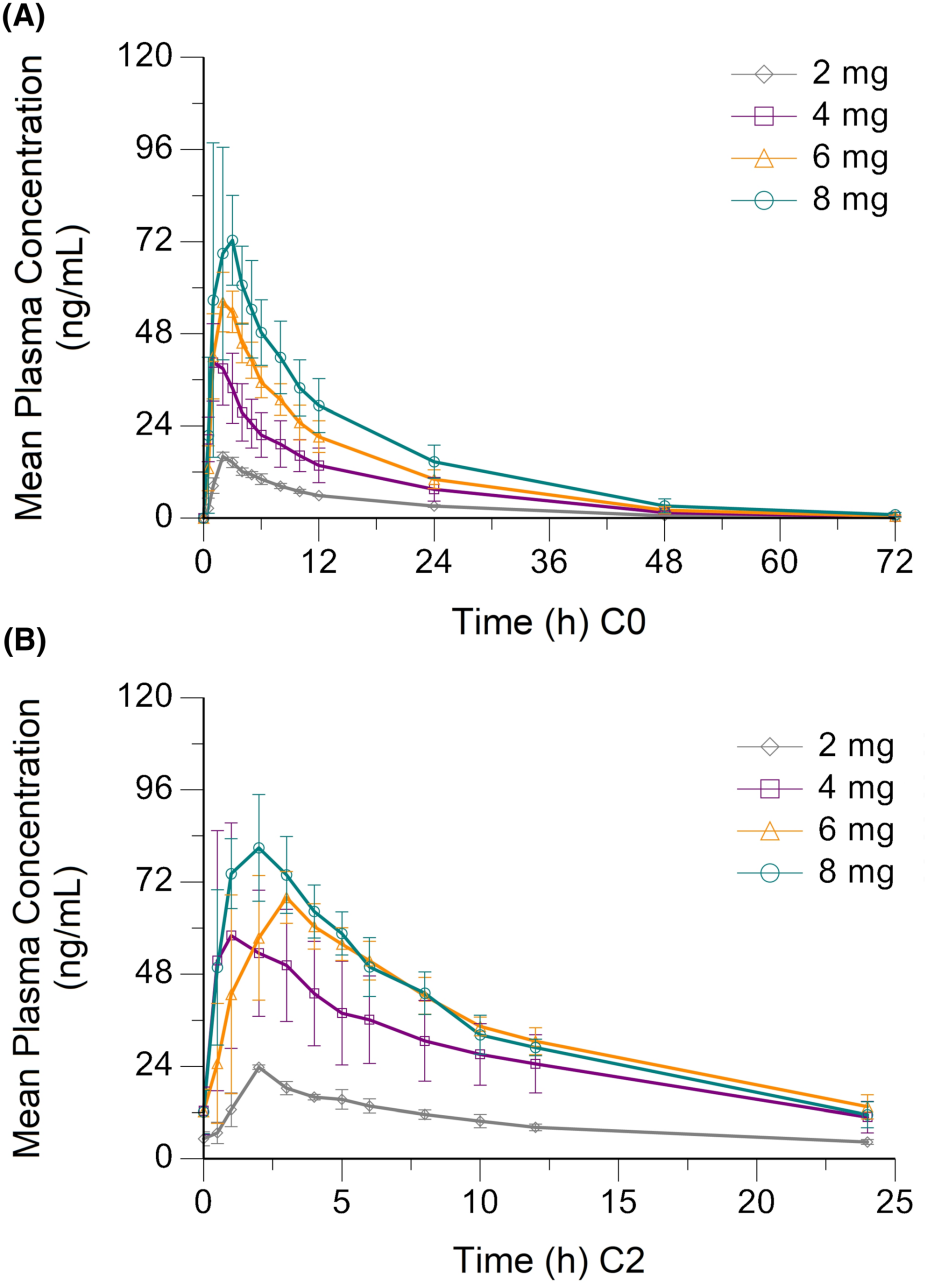
Concentration–time curves in the 2, 4, 6, and 8 mg QD groups after single‐dose administration in Cycle 0 (A) and multiple‐dose administration in Cycle 2 (B).

Following a single‐dose oral administration of 2, 4, 6, or 8 mg in patients with ABC, HS‐10352 was rapidly absorbed. The median times to peak (*T*_max_) ranged from 1.0 to 2.5 h and the mean elimination half‐life (*t*_1/2_) values ranged from 11.2 to 15.2 h across the tested dose levels (Figure 2A and Table S5). In the dose range of 2 to 8 mg, all mean maximum concentration (*C*_max_) values (range, 16.09–80.25 ng/mL), mean concentrations from 0 to the last measurable (AUC_0‐t_) (range, 217.19–1094.67 h × ng/mL) and mean areas under the blood concentration–time curves (AUC_0‐∞_) (range, 218.87–1096.84 h × ng/mL) showed proportionally increasing trends based on dose, exhibiting linear pharmacokinetic characteristics (Tables S5, S6).

After multiple‐dose administrations, HS‐10352 reached the steady state by Day 8, with mean steady‐state maximum blood concentration (*C*_ss,max_) values of 23.76 to 81.79 ng/mL and mean areas under the concentration–time curves at the steady state (AUC_ss_) of 231.26 to 851.67 h × ng/mL (Table S7). Both *C*_ss,max_ and AUC_ss_ increased with drug dose in a dose‐dependent manner (Table S8). The AUC_ss_ of the 6 mg QD group was close to that of the 8 mg QD group, and the exposure dose showed a trend of saturation (Figure 2B). In addition, the minimum concentrations at the steady state (*C*_ss,min_) were similar in the 4, 6, and 8 mg QD groups, indicating that increasing the dose did not further increase *C*_ss,min_ with an once‐daily dosing regimen (Table S7).

### 3.4 Efficacy

**FIGURE 3:**
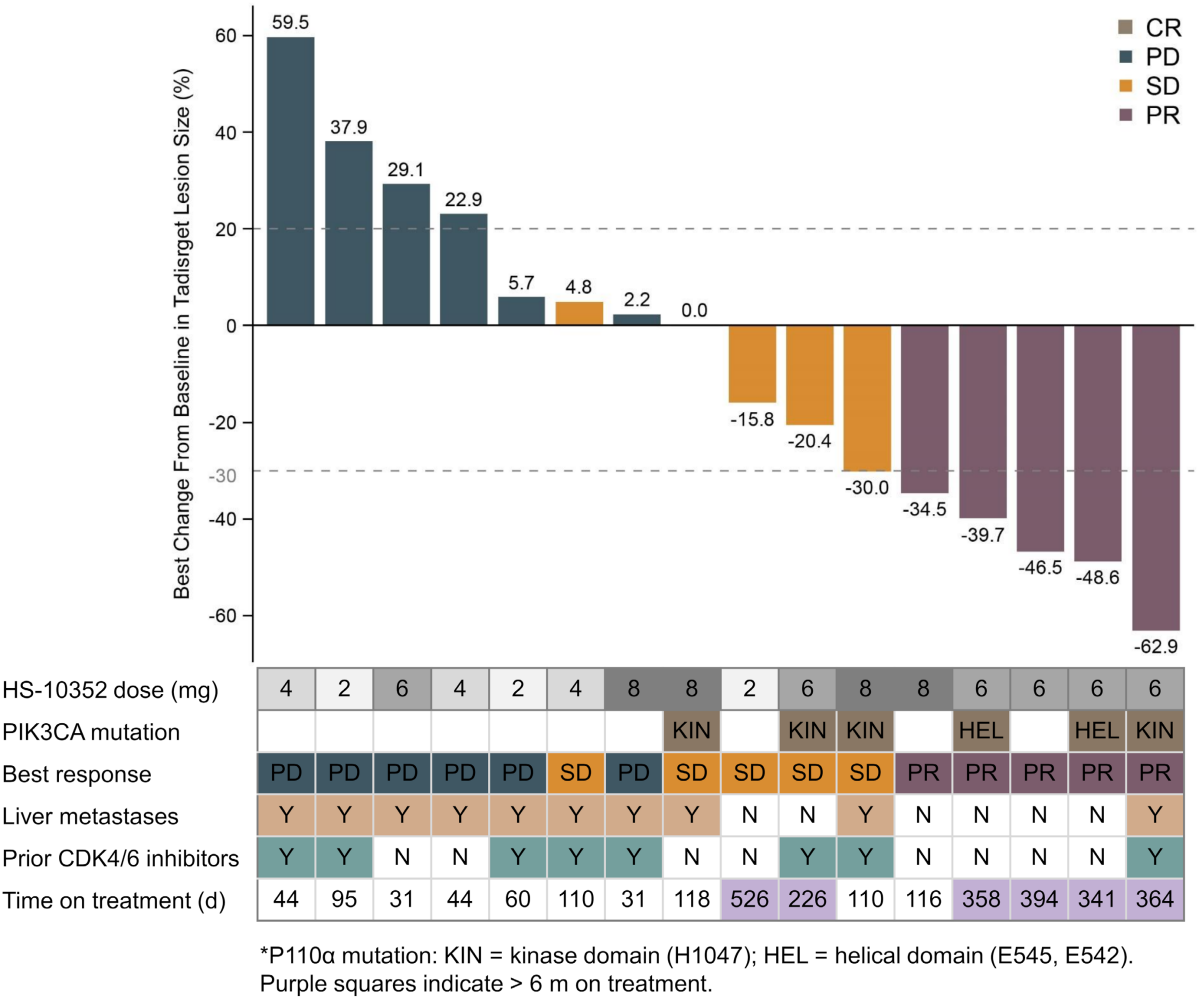
Waterfall plot for tumor response in individual patients. CR, complete response; PR, partial response; SD, stable disease; PD, progressive disease; Y, yes; N, no.

Five of the 18 patients had PR (4 and 1 in the 6 mg and 8 mg QD groups, respectively), with an ORR of 27.8% (95% CI, 9.7%–53.5%), a DCR of 55.6% (95% CI, 30.8%–78.5%) and a median PFS of 3.9 (95% CI, 2.0‐NA) months. In the 6 mg QD group, ORR and DCR were 66.7% (95% CI, 22.3%–95.7%) and 83.3% (95% CI, 35.9%–99.6%), respectively, and median PFS was not reached at the time of data cut‐off (Table 3). The median DoR was not reached in the 6 mg QD or 8 mg QD group (Table S9). The waterfall plot for tumor response in individuals is shown in Figure 3.

**TABLE 3:** Efficacy data.

	2 mg QD	4 mg QD	6 mg QD	8 mg QD	Total
	(*N* = 3)	(*N* = 3)	(*N* = 6)	(*N* = 6)	(*N* = 18)
Tumor response, *n* (%)					
CR	0	0	0	0	0
PR	0	0	4 (66.7)	1 (16.7)	5 (27.8)
SD	1 (33.3)	1 (33.3)	1 (16.7)	2 (33.3)	5 (27.8)
PD	2 (66.7)	2 (66.7)	1 (16.7)	1 (16.7)	6 (33.3)
NE	0	0	0	2 (33.3)	2 (11.1)
ORR, % (95% CI)	0 (0, 70.8)	0 (0, 70.8)	66.7 (22.3, 95.7)	16.7 (0.4, 64.1)	27.8 (9.7, 53.5)
DCR, % (95% CI)	33.3 (0.8, 90.6)	33.3 (0.8, 90.6)	83.3 (35.9, 99.6)	50.0 (11.8, 88.2)	55.6 (30.8, 78.5)
PFS, months, median (95% CI)	2.1 (2.0, NA)	2.3 (1.7, NA)	NA (1.2, NA)	3.8 (1.2, NA)	3.9 (2.0, NA)

Abbreviations: CI, confidence interval; CR, complete response; DCR, disease control rate; NA, not available; NE, not evaluable; ORR, objective response rate; PD, progressive disease; PR, partial response; PFS, progression‐free survival; SD, stable disease.

For the 9 patients with PIK3CA mutated tumors confirmed by the central laboratory and/or previous medical records, ORR and DCR were 44.4% (95% CI, 13.7%–78.8%) and 77.8% (95% CI, 40.0%–97.2%), respectively. The median PFS was 11.1 (95% CI, 2.1‐NA) months (Table S10).

For the six patients with PIK3CA mutated tumors confirmed by the central laboratory, ORR, DCR, and DoR were 50.0% (95% CI, 11.8%–88.2%), 100.0% (95% CI, 54.1%–100.0%), and NA (95% CI, 9.1‐NA) months, respectively (Table S11). Of these six patients, four were administered HS‐10352 at 6 mg QD, showing an ORR of 75.0% (95% CI, 19.4%–99.4%) and a DCR of 100.0% (95% CI, 39.8%–100.0%). The median PFS was NA (95% CI, 11.1‐NA) months and median DoR was NA (95% CI, 9.1‐NA) months. The spider plot for these 4 patients is presented in Figure S2.

For the 10 patients with PIK3CA wild‐type tumors confirmed by the central laboratory, the ORR and DCR were 10.0% (95% CI, 0.3%–44.5%) and 20.0% (95% CI, 2.5%–55.6%), respectively (Table S12). The median DoR was NA (95% CI, NA‐NA) and median PFS was 2.1 (95% CI, 1.2‐NA) months (Figure S3).

Of the five patients with confirmed PR, three had PR at the first follow‐up visit for tumor assessment on Day 1 of Cycle 3 (C3D1), and the other two had PR at the third follow‐up visit on C7D1. For example, one PIK3CA‐positive patient in the 6 mg QD group had PR on C3D1, and the target lesion continued to shrink in subsequent follow‐up visits, maintaining PR as of the data cut‐off date (C15D1). Another PIK3CA‐positive patient in the 6 mg QD group had target lesion shrinkage on C3D1 and had PR on C7D1, maintaining PR as of the data cut‐off date (C13D1) (Figure S4).

## 4 DISCUSSION

The efficacy of PI3K inhibitors has been confirmed in randomized phase 3 trials.^9^, ^14^, ^15^ However, their safety remains a concern for patients with breast cancer. HS‐10352 is a novel oral selective PI3Kα inhibitor. This phase 1 dose‐escalation study evaluated the safety, tolerability, pharmacokinetics, and preliminary antitumor activity of HS‐10352 in patients with HR‐positive, HER2‐negative ABC. As anticipated, hyperglycemia was the most common TRAE, occurring in 88.9% of patients. The MTD was determined to be 6 mg once‐daily. Notably, a promising tumor response was observed in the 6 mg QD group, particularly among patients with PIK3CA mutated tumors.

The safety profile of pan‐PI3K inhibitors has been a limiting factor for their use in clinical practice.^16^ HS‐10352 selectively inhibits PI3Kα kinase activity, thereby minimizing many of the serious side effects commonly associated with the inhibition of PI3Kβ, δ, and γ. DLT, specifically hyperglycemia, occurred in two patients at a dose of 8 mg QD, while no DLTs were observed at 6 mg QD. Grade 3 or higher TRAEs, SAEs, and TRAEs leading to treatment discontinuation were reported in 5 (27.8%), 1 (5.6%), and 1 (5.6%) out of 18 patients, respectively. Notably, most of these adverse events were observed in the 8 mg QD group. In the 6 mg QD group, two grade 3 or higher TRAEs (weight loss) were reported. The most common TRAEs in this group were hyperglycemia, weight loss, diarrhea, and hyponatremia. The safety profile of HS‐10352 aligns well with those of other PI3Kα inhibitors used as monotherapies in previous reports,^17^, ^18^, ^19^ with a few exceptions: rash, pruritus, stomatitis, mucositis, and mood alterations were relatively uncommon with HS‐10352 treatment. Notably, the CTCAE 5.0 criteria were used in the current study to evaluate the severity of hyperglycemia, as opposed to the CTCAE 4.03 criteria^20^ used in previous studies. Using CTCAE 4.03 would have resulted in a grade ≥3 hyperglycemia incidence of 16.7% (3/18). HS‐10352‐associated rash was mild (grades 1–2) and did not necessitate dose adjustments or the use of oral steroids. These findings suggest that an MTD of 6 mg QD is both safe and manageable. The incidence, types, and severity of AEs were higher in the 6 mg and 8 mg QD groups compared with the 2 mg and 4 mg QD groups, indicating a dose‐dependent increase in adverse events. Overall, this dose‐escalation study suggests that HS‐10352 has a manageable safety profile.

HS‐10352 exhibited favorable pharmacokinetic characteristics. According to the concentration–time curves, an increase in dosage did not correspondingly elevate the trough concentration after multiple administrations. Notably, the plasma level of HS‐10352 at an 8 mg dose appeared to plateau, suggesting dose saturation.

PIK3CA mutations often result in hyperactivation of the PI3K/AKT/mTOR pathway, which is associated with oncogenesis, tumor development, and resistance to endocrine therapy in HR‐positive, HER2‐negative ABC.^21^, ^22^, ^23^, ^24^, ^25^ As such, the inhibition of PI3K signaling presents a promising therapeutic avenue.^26^, ^27^, ^28^, ^29^, ^30^ In the current study, all enrolled patients with HR‐positive, HER2‐negative ABC that exhibited insufficient responses to standard of care, or had no access to, or demonstrated intolerance to standard therapies were heavily pre‐treated. Nevertheless, the ORR of HS‐10352 monotherapy for patients with PIK3CA mutated tumors stood at an encouraging 50.0%, and the median PFS was 11.1 months. More impressively, among patients with PIK3CA mutated tumors in the 6 mg QD group, the ORR reached 75.0%. Furthermore, the median PFS had yet to be reached at a median follow‐up of 12.9 months (range: 7.5–13.1 months). These findings compare favorably to existing literature on other therapies targeting the PI3K/AKT/mTOR pathway.^17^, ^18^, ^19^, ^31^, ^32^ Typically, the single‐agent efficacy of this class of drugs is moderate. For instance, alpelisib, the only Food and Drug Administration (FDA)‐approved PI3Kα inhibitor for breast cancer, had a mere 4.3% ORR when used as a monotherapy in ER‐positive, HER2‐negative breast cancer patients.^19^ GDC‐0077, another selective PI3Kα inhibitor, fared better as a single agent, having a confirmed ORR of 20% in PIK3CA‐mutant, HR‐positive, HER2‐negative breast cancer patients.^33^ Capivasertib, a pan‐AKT inhibitor, reached an ORR of just 4% in PIK3CA‐mutant breast cancer patients.^31^ Among the 10 patients with central laboratory confirmed PIK3CA wild‐type tumors, the ORR was 10% and the median PFS was 2.1 months with HS‐10352, numerically inferior to the results of those with PIK3CA mutated tumors (ORR, 50%; median PFS, 11.1 months). This was consistent with our preclinical results showing that HS‐10352 had a high selectivity for the cell models harboring mutations, indicating a worse response in patients with PIK3CA wild‐type tumors compared with those with PIK3CA mutated tumors.

Given that inhibitors of the PI3K/AKT/mTOR pathway can mitigate endocrine therapy resistance in HR‐positive, HER2‐negative ABC, combination therapy emerges as a compelling strategy. Agents like fulvestrant can notably enhance the sensitivity of ER‐positive tumors to PI3Kα inhibition, thereby facilitating substantial tumor regression in vivo.^34^ For example, the SOLAR‐1 trial investigated the combination of alpelisib and fulvestrant in patients with PIK3CA‐mutant, HR‐positive, HER2‐negative breast cancer. The study reported an ORR of 26.6%, compared with 12.6% in the placebo plus fulvestrant group. Additionally, the median PFS was 11.0 months, as opposed to 5.7 months in the placebo group. Similarly, the CAPItello‐291 trial evaluated the efficacy of fulvestrant, with or without capivasertib, in HR‐positive, HER2‐negative breast cancer patients who had altered AKT pathways (nearly 75% were PIK3CA‐mutant). The study reported an ORR of 28.8% versus 9.7% and a median PFS of 7.3 versus 3.1 months, respectively.^35^ Currently, a clinical trial is underway to assess the safety, tolerability, pharmacokinetics, and efficacy of HS‐10352 in combination with fulvestrant in patients with PIK3CA‐mutant, HR‐positive, HER2‐negative advanced breast cancer (NCT05504213). Given the relatively favorable efficacy data from HS‐10352 monotherapy, especially when compared with molecules in the same class such as alpelisib and capivasertib, there is reason to be optimistic that this combination could represent a promising therapeutic option for addressing this unmet clinical need.

Despite exhibiting a higher response rate compared with other PI3K/AKT/mTOR pathway inhibitors used as monotherapies in previous reports,^17^, ^18^, ^19^, ^31^, ^32^ the findings of this dose‐escalation trial have some limitations. First, the number of subjects enrolled to date is relatively small. Second, a longer follow‐up period is needed to obtain the final survival outcomes. Third, the number of patients with PIK3CA mutated tumors was small. Given the poor response in patients with PIK3CA wild‐type tumors, further assessments of HS‐10352 will focus on patients with PIK3CA mutated tumors. Finally, no comprehensive next‐generation sequencing data was available in our study, which limited our ability to explore the potential influence of specific genetic alterations, such as PTEN/AKT mutations, on treatment efficacy and outcomes. These will be investigated in the phase 1b study of HS‐10352 (NCT05504213).

## 5 CONCLUSION

HS‐10352, administered at 6 mg once‐daily, was well‐tolerated in patients with HR‐positive, HER2‐negative ABC and showed promising antitumor activity, particularly in individuals with PIK3CA mutated tumors. These findings lend support to the further clinical development of HS‐10352, including its potential use in combination therapies such as endocrine therapy.

## AUTHOR CONTRIBUTIONS

**Quchang Ouyang:** Methodology (equal); writing – original draft (equal); writing – review and editing (equal). **Ying Wang:** Methodology (equal); writing – original draft (equal); writing – review and editing (equal). **Jian Zhang:** Methodology (equal); writing – original draft (equal); writing – review and editing (equal). **Qiong Wu:** Conceptualization (equal); supervision (equal); writing – original draft (equal); writing – review and editing (equal). **Hongying Wei:** Formal analysis (equal); writing – original draft (equal); writing – review and editing (equal). **Chuan Li:** Formal analysis (equal); writing – original draft (equal); writing – review and editing (equal). **Xiaoling Qian:** Data curation (equal); writing – original draft (equal); writing – review and editing (equal). **Xichun Hu:** Conceptualization (equal); methodology (equal); supervision (equal); writing – original draft (equal); writing – review and editing (equal).

## CONFLICT OF INTEREST STATEMENT

All authors declare no financial or non‐financial conflicts of interests.

## ETHICS STATEMENT

The study protocol was registered at ClinicalTrials.gov (NCT04631835) and approved by the ethics committee of Fudan University Shanghai Cancer Center (No. 2006218–20). All patients provided signed informed consent prior to enrolment.

## Supporting information


**Data S1:** Supporting Information.

## Data Availability

All data generated or analyzed in this study are included in the published article and its supplementary information files.
