# Supplementary material for: HS‐10352 in hormone receptor‐positive, HER2‐negative advanced breast cancer: A phase 1 dose‐escalation trial
Source: Cancer Med. 2023 Dec 1;12(24):21849–60. doi: 10.1002/cam4.6755 (PMC10757118; doi:10.1002/cam4.6755)
Supplement: Supplementary file 1 — Data S1: Supporting Information. [file CAM4-12-21849-s001.docx]

**Supplementary materials**

**Administration of HS-10352**

HS-10352 was administered as a whole tablet with approximately 240 mL of drinking water of ambient. The subjects fasted from 1 hour before to 2 hours after drug intake. The patients received HS-10352 continuously until disease progression or the occurrence of any other criteria for treatment discontinuation.

**Management of toxicity**

When several AEs occurred simultaneously, dose adjustment was decided based on the most severe AE. When grade ≥3 toxicity occurred, HS-10352 was temporarily discontinued, and supportive treatment was provided as needed according to the local practice or guidelines. In case grade ≥3 toxicity returned to grade ≤1 or baseline within 14 days, the dose was reduced to a lower level (or maintained at the lowest level); otherwise, HS-10352 was permanently discontinued. Once the dose was reduced, it was not allowed to be increased to the previous level. Patients were suggested to withdraw from the study if not tolerating the lowest dose. In patients with permanent discontinuation of the study drug, follow-up was continued until toxicity was relieved. Dose interruption did not affect the evaluation of treatment response.

**Laboratory tests and electrocardiography**

Samples were collected for blood routine, urine routine, blood biochemistry, coagulation function, fasting blood glucose, insulin, and C-peptide tests in the screening period, on day 1 in cycle 0 (C0D1), C1D1, C1D8, C1D15, C1D22, C2D1 and the first day of each subsequent cycle, and at the end of treatment. Electrocardiograms were obtained at the same time points mentioned above. HbA1c was assessed in the screening period, on C1D1 and every two cycles thereafter, and at the end of treatment. Females of childbearing age were tested for human chorionic gonadotropin (hCG) levels in the screening period, on C1D1 and every cycle thereafter, and at the end of treatment.

Evaluation of antitumor activity

Treatment response was assessed every 2 cycles (8 ±1 weeks) since the first dose (C1D1) until disease progression according to RECIST v1.1 or any other criteria for treatment discontinuation were met.

Confirmation of a CR/PR implied a first evaluation of a CR/PR and confirmation by repeated imaging at least 4 weeks later. The last evaluable data in the absence of disease progression were included in ORR evaluation. Any CR or PR occurring after treatment discontinuation and receipt of further anticancer therapy was not included in ORR determination. Disease control rate (DCR) was defined as the proportion of patients having a CR, a PR, or stable disease (SD).

PFS was defined as the time between enrollment and disease progression or death from any cause.

Duration of remission (DoR) was defined as the time between the first CR or PR and the date of disease progression or death from any cause.

Overall survival (OS) was assessed based on the date of the first dose of the study drug and survival status at the data cut-off date. OS was defined as the interval between the date of the first dosing and the date of death from any cause.

**Pharmacokinetic assessment**

Venous blood samples for the determination of plasma HS-10352 concentrations were collected at the time points listed in Table S2. When dose interruption occurred during PK sampling, discussions with Hansoh were carried out to determine whether the PK sampling schedule needed adjustment. During the study period, the subsequent PK sample collection times were allowed to be adjusted according to the interim PK results. A 5-minute window was allowed for samples obtained at 30 minutes, with a 10-minute window for samples obtained at 1 to 10 hours, a 1‑hour window for samples obtained at 12 hours and a 2‑hour window for samples obtained at 24 to 120 hours.

Pharmacokinetic parameters for the single-dose administration included the maximum concentration (C_max_), time to peak (T_max_), concentration from 0 to 24 hours (AUC_0-24_), concentration from 0 to the last measurable (AUC_0-t_), and area under the blood concentration-time curve (AUC_0-∞_). Pharmacokinetic parameters for the multiple-dose administration included steady-state maximum blood concentration (C_ss,max_) and the area under the blood concentration-time curve (AUC_ss_) within one dosing interval at the steady state.

**Supplementary table 1**. Participating centers

| No. | Name of the center | Number of patients screened | Number of patients enrolled |
| --- | --- | --- | --- |
| 1 | Fudan University Shanghai Cancer Center | 10 | 4 |
| 2 | Sun Yat-sen Memorial Hospital of Sun Yat-sen University | 10 | 7 |
| 3 | Hunan Cancer Hospital | 11 | 7 |

**Supplementary table 2.** Adverse events and treatment-related adverse events

| Event, n (%) | 2 mg QD | 4 mg QD | 6 mg QD | 8 mg QD | Total |
| --- | --- | --- | --- | --- | --- |
|  | (N=3) | (N=3) | (N=6) | (N=6) | (N=18) |
| Any AEs | 3 (100) | 3 (100) | 6 (100) | 6 (100) | 18 (100) |
| Any TRAEs | 3 (100) | 3 (100) | 6 (100) | 6 (100) | 18 (100) |
| Grade ≥3 AEs | 0 | 0 | 2 (33.3) | 3 (50.0) | 5 (27.8) |
| Grade ≥3 TRAEs | 0 | 0 | 2 (33.3) | 3 (50.0) | 5 (27.8) |
| DLT | 0 | 0 | 0 | 2 (33.3) | 2 (11.1) |
| SAEs | 0 | 0 | 0 | 1 (16.7) | 1 (5.6) |
| TRSAEs | 0 | 0 | 0 | 1 (16.7) | 1 (5.6) |
| AEs leading to dose reduction | 0 | 0 | 1 (16.7) | 2 (33.3) | 3 (16.7) |
| TRAEs leading to dose reduction | 0 | 0 | 1 (16.7) | 2 (33.3) | 3 (16.7) |
| AEs leading to dose interruption | 0 | 1 (33.3) | 0 | 0 | 1 (5.6) |
| TRAEs leading to dose interruption | 0 | 1 (33.3) | 0 | 0 | 1 (5.6) |
| AEs leading to discontinuation | 0 | 0 | 0 | 1 (16.7) | 1 (5.6) |
| TRAEs leading to discontinuation | 0 | 0 | 0 | 1 (16.7) | 1 (5.6) |
| AEs leading to death | 0 | 0 | 0 | 1 (16.7) | 1 (5.6) |
| TRAEs leading to death | 0 | 0 | 0 | 0 | 0 |

AE, adverse event; TRAE, treatment-related adverse event; DLT, dose-limiting toxicity; SAE, serious adverse event; TRSAE, treatment-related serious adverse event.

**Supplementary table 3.** Drug exposure in patients during the repeat dosing phase

|  | 2 mg QD | 4 mg QD | 6 mg QD | 8 mg QD | Total |
| --- | --- | --- | --- | --- | --- |
|  | (N=3) | (N=3) | (N=6) | (N=6) | (N=18) |
| Days of exposure (days) | 226.0±259.53 | 66.0±39.84 | 288.8±140.73 | 72.8±46.48 | 169.2±159.21 |
| Days of continuous exposure (days) | 226.0±259.53 | 65.0±38.11 | 284.7±137.54 | 71.2±47.72 | 167.1±157.82 |
| Cumulative exposure dose (mg) | 452.0±519.06 | 260.0±152.42 | 1632.0±793.67 | 524.3±412.74 | 837.4±782.49 |
| Mean daily exposure dose (mg) | 2.0±0.00 | 4.0±0.06 | 5.7±0.65 | 6.7±1.91 | 5.1±2.04 |
| Actual daily exposure dose (mg) | 2.0±0.00 | 4.0±0.00 | 5.8±0.53 | 6.9±1.65 | 5.2±2.01 |

**Supplementary table 4.** Schedule of blood sampling for PK analysis

| **Time relative to dose** | **Single dosing** | | | | | | **Multiple dosing** | | | | | |
| --- | --- | --- | --- | --- | --- | --- | --- | --- | --- | --- | --- | --- |
|  | **Cycle 0** | | | | | | **Cycle 1** | | | | **Cycle 2** | |
|  | **D1** | **D2** | **D3** | **D4** | **D5** | **D6** | **D1** | **D8** | **D15** | **D22** | **D1** | **D2** |
| Sampling window (day) | 0 |  |  |  |  |  | 0 | ±1 | ±2 | ±2 | ±2 | ±2 |
| Predose | ╳ |  |  |  |  |  | ╳ | ╳ | ╳ | ╳ | ╳ |  |
| 0.5h±5min | ╳ |  |  |  |  |  |  |  |  |  | ╳ |  |
| 1h±10min | ╳ |  |  |  |  |  |  |  |  |  | ╳ |  |
| 2h±10min | ╳ |  |  |  |  |  |  |  |  |  | ╳ |  |
| 3h±10min | ╳ |  |  |  |  |  |  |  |  |  | ╳ |  |
| 4h±10min | ╳ |  |  |  |  |  |  |  |  |  | ╳ |  |
| 5h±10min | ╳ |  |  |  |  |  |  |  |  |  | ╳ |  |
| 6h±10min | ╳ |  |  |  |  |  |  |  |  |  | ╳ |  |
| 8h±10min | ╳ |  |  |  |  |  |  |  |  |  | ╳ |  |
| 10h±10min | ╳ |  |  |  |  |  |  |  |  |  | ╳ |  |
| 12h±1h | ╳ |  |  |  |  |  |  |  |  |  | ╳ |  |
| 24h±2h |  | ╳ |  |  |  |  |  |  |  |  |  | ╳*  (Predose) |
| 48h±2h |  |  | ╳ |  |  |  |  |  |  |  |  |  |
| 72h±2h |  |  |  | ╳ |  |  |  |  |  |  |  |  |
| 96h±2h |  |  |  |  | ╳ |  |  |  |  |  |  |  |
| 120h±2h |  |  |  |  |  | ╳ |  |  |  |  |  |  |

* For patients receiving multiple-dose administration, there was no sampling point at 24h on day 2 in cycle 2.

Supplementary table 5. PK parameters for each dose group of HS-10352 after single dose administration in phase I dose-escalation period-PKS

| **PK Parameter (unit)** | | **2 mg QD**  **(N=3)** | **4 mg QD**  **(N=3)** | **6 mg QD**  **(N=6)** | **8 mg QD**  **(N=6)** |
| --- | --- | --- | --- | --- | --- |
| C_max_  (ng/mL) | Mean±SD | 16.09±1.12 | 42.14±9.41 | 57.30±6.39 | 80.25±21.54 |
|  | Gmean (%CV_b_) | 16.06 (6.89) | 41.45 (22.54) | 57.01 (10.98) | 78.22 (24.22) |
| T_max_ (h) | Median (Min, Max) | 2.0 (2.0,2.0) | 1.0 (1.0,2.0) | 2.5 (1.9,3.0) | 2.0 (0.9,3.0) |
| AUC_0-t_  (h*ng/mL) | Mean±SD | 217.19±3.08 | 533.60±154.71 | 790.65±126.43 | 1094.67±238.99 |
|  | Gmean (%CV_b_) | 217.17 (1.42) | 517.60 (31.59) | 782.45 (15.82) | 1074.07 (21.39) |
| AUC_0-∞_  (h*ng/mL) | Mean±SD | 218.87±3.15 | 535.00±155.19 | 792.66±127.61 | 1096.84±238.99 |
|  | Gmean (%CV_b_) | 218.86 (1.45) | 518.96 (31.58) | 784.34 (15.91) | 1076.26 (21.36) |
| t_1/2_ (h) | Mean±SD | 11.2±2.1 | 15.2±4.1 | 13.9±4.1 | 12.4±2.3 |
| CL/F (L/h) | Mean±SD | 9.14±0.13 | 7.96±2.54 | 7.73±1.20 | 7.57±1.55 |
| V_d_/F (L) | Mean±SD | 148.1±29.2 | 168.6±38.8 | 153.5±46.2 | 135.3±33.3 |
| λ_z_ (1/h) | Mean±SD | 0.063±0.011 | 0.048±0.011 | 0.053±0.015 | 0.057±0.009 |
| MRT (h) | Mean±SD | 15.32±2.11 | 15.20±0.47 | 14.88±2.18 | 15.65±3.15 |

C_max_, maximum concentration; T_max_, time to peak concentration; AUC_0-t_, area under concentration-time curve from 0 to the last measurable; AUC_0-∞_, area under concentration-time curve from 0 to infinity; t_1/2_, half-life; CL/F, apparent clearance; V_d_/F, apparent volume of distribution; λ_z_, terminal phase elimination rate constant; MRT, mean residence time.

Supplementary table 6. Correlations of HS-10352 PK parameters with the dose administered after a single dose in phase I dose-escalation period-PKS

| **Analyte** | **PK Parameters (Unit)** | **Dosage Range** | **N** | **Estimated Mean Slope（b）** | **Lower Limit of 90%CI** | **Upper Limit of 90%CI** |
| --- | --- | --- | --- | --- | --- | --- |
| HS-10352 | C_max_ (ng/mL) | 2~8 mg | 18 | 1.1164 | 0.9641 | 1.2687 |
|  | AUC_0-t_ (h*ng/mL) | 2~8 mg | 18 | 1.1440 | 0.9859 | 1.3021 |
|  | AUC_0-∞_ (h*ng/mL) | 2~8 mg | 18 | 1.1401 | 0.9819 | 1.2982 |

N, sample size; CI, confidence interval; C_max_, maximum concentration; AUC_0-t_, area under concentration-time curve from 0 to the last measurable; AUC_0-∞_, area under concentration-time curve from 0 to infinity.

Supplementary table 7. PK parameters of each dose group of HS-10352 after multiple dosing in phase I dose-escalation period-PKS

| **PK Parameter (unit)** | | **2 mg QD**  (N=3) | **4 mg QD**  (N=3) | **6 mg QD**  (N=6) | **8 mg QD**  (N=3) |
| --- | --- | --- | --- | --- | --- |
| C_ss,max_  (ng/mL) | Mean±SD | 23.76±0.58 | 60.31±26.48 | 71.88±9.18 | 81.79±12.35 |
|  | Gmean (%CV_b_) | 23.75 (2.41) | 56.73 (44.17) | 71.39 (12.81) | 81.13 (15.96) |
| C_ss,min_  (ng/mL) | Mean±SD | 4.31±0.62 | 10.77±4.12 | 11.59±5.37 | 11.66±5.13 |
|  | Gmean (%CV_b_) | 4.28 (14.88) | 10.22 (42.02) | 8.95 (137.20) | 10.71 (58.07) |
| C_ss,av_  (ng/mL) | Mean±SD | 9.64±0.83 | 27.21±9.17 | 33.69±3.08 | 35.49±4.67 |
|  | Gmean (%CV_b_) | 9.61 (8.51) | 26.20 (34.63) | 33.57 (9.24) | 35.29 (12.86) |
| T_ss,max_ (h) | Median (Min, Max) | 2.0 (2.0,2.1) | 1.0 (1.0, 2.0) | 3.0 (0.9, 4.0) | 2.0 (1.0, 2.0) |
| AUC_ss_  (h*ng/mL) | Mean±SD | 231.26±19.78 | 653.04±220.15 | 808.54±73.90 | 851.67±112.27 |
|  | Gmean (%CV_b_) | 230.70 (8.49) | 628.80 (34.63) | 805.70 (9.24) | 846.93 (12.88) |

C_ss,max_, maximum concentration at steady state; C_ss,min_, minimum concentration at steady state; C_ss,av_, average concentration at steady state; T_ss,max_, time to peak concentration at steady state; AUC_ss_, area under concentration-time curve during dosing interval.

Supplementary table 8. Correlations of HS-10352 PK parameters with administered dose after multiple dosing in phase I dose-escalation period-PKS

| **Analyte** | **Dosing Frequency** | **PK Parameters (unit)** | **Dosage Range** | **N** | **Estimated Mean Slope (b)** | **Lower Limit of 90%CI** | **Upper Limit of 90%CI** |
| --- | --- | --- | --- | --- | --- | --- | --- |
| HS-10352 | QD | C_ss,max_ (ng/mL) | 2~8 mg | 15 | 0.9035 | 0.6950 | 1.1120 |
|  |  | AUC_ss_ (h*ng/mL) | 2~8 mg | 15 | 0.9812 | 0.7777 | 1.1846 |

N, sample size; CI, confidence interval; C_ss,max_, maximum concentration at steady state; AUC_ss_, area under concentration-time curve during dosing interval.

**Supplementary table 9.** Duration of response for all responders

|  | 6 mg QD | 8 mg QD | Total |
| --- | --- | --- | --- |
|  | (N=4) | (N=1) | (N=5) |
| DoR, n (%) |  |  |  |
| <3 months | 0 | 1 (100) | 1 (20.0) |
| 3-6 months | 1 (25.0) | 0 | 1 (20.0) |
| 6-9 months | 1 (25.0) | 0 | 1 (20.0) |
| ≥9 months | 2 (50.0) | 0 | 2 (40.0) |
| Median DoR (95%CI), months | NA (9.1, NA) | NA (NA, NA) | NA (9.1, NA) |

DoR, duration of response; CI, confidence interval; NA, not available.

**Supplementary table 10.** Efficacy in patients with PIK3CA mutated tumors confirmed by the central laboratory or past medical records

|  | 2 mg QD | 6 mg QD | 8 mg QD | Total |
| --- | --- | --- | --- | --- |
|  | (N=1) | (N=4) | (N=4) | (N=9) |
| Tumor response, n (%) |  |  |  |  |
| CR | 0 | 0 | 0 | 0 |
| PR | 0 | 3 (75.0) | 1 (25.0) | 4 (44.4) |
| SD | 0 | 1 (25.0) | 2 (50.0) | 3 (33.3) |
| PD | 1 (100) | 0 | 0 | 1 (11.1) |
| NE | 0 | 0 | 0 | 0 |
| ORR, n (%), (95%CI) | 0  (0, 97.5) | 3 (75.0)  (19.4, 99.4) | 1 (25.0)  (0.6, 80.6) | 4 (44.4)  (13.7, 78.8) |
| DCR, n (%), (95%CI) | 0  (0, 97.5) | 4 (100.0)  (39.8, 100.0) | 3 (75.0)  (19.4, 99.4) | 7 (77.8)  (40.0, 97.2) |
| Median PFS (95%CI), months | 2.1 (NA, NA) | NA (11.1, NA) | 3.9 (2.2, NA) | 11.1 (2.1, NA) |

CR, complete response; PR, partial response; SD, stable disease; PD, progressive disease; NE, not evaluable; ORR, objective response rate; CI, confidence interval; DCR, disease control rate; PFS, progression-free survival; NA, not available; DoR, duration of response.

**Supplementary table 11.** Efficacy for patients with PIK3CA mutated tumors confirmed by the central laboratory only

|  | 6 mg/d | 8 mg/d | Total |
| --- | --- | --- | --- |
|  | (N=4) | (N=2) | (N=6) |
| Tumor response, n (%) |  |  |  |
| CR | 0 | 0 | 0 |
| PR | 3 (75.0) | 0 | 3 (50.0) |
| SD | 1 (25.0) | 2 (100.0) | 3 (50.0) |
| PD | 0 | 0 | 0 |
| NE | 0 | 0 | 0 |
| ORR, n (%), (95%CI) | 3 (75.0) (19.4-99.4) | 0  (0, 84.2) | 3 (50.0)  (11.8, 88.2) |
| DCR, n (%), (95%CI) | 4 (100.0)  (39.8, 100.0) | 2 (100.0)  (15.8, 100.0) | 6 (100.0)  (54.1, 100.0) |
| Median PFS (95%CI), months | NA (11.1, NA) | 3.9 (3.8, NA) | 11.1 (3.8, NA) |
| Median DoR (95%CI), months | NA (9.1, NA) | - | NA (9.1, NA) |

CR, complete response; PR, partial response; SD, stable disease; PD, progressive disease; NE, not evaluable; ORR, objective response rate; CI, confidence interval; DCR, disease control rate; PFS, progression-free survival; NA, not available; DoR, duration of response.

**Supplementary table 12.** Efficacy for patients with PIK3CA wild-type tumors confirmed by the central laboratory

|  | 2 mg/d | 4 mg/d | 6 mg/d | 8 mg/d | Total |
| --- | --- | --- | --- | --- | --- |
|  | (N=3) | (N=2) | (N=2) | (N=3) | (N=10) |
| Tumor response, n (%) |  |  |  |  |  |
| CR | 0 | 0 | 0 | 0 | 0 |
| PR | 0 | 0 | 1 (50.0) | 0 | 1 (10.0) |
| SD | 1 (33.3) | 0 | 0 | 0 | 1 (10.0) |
| PD | 2 (66.7) | 2 (100.0) | 1 (50.0) | 1 (33.3) | 6 (60.0) |
| NE | 0 | 0 | 0 | 2 (66.7) | 2 (20.0) |
| ORR, n (%), (95%CI) | 0  (0.0, 70.8) | 0  (0.0, 84.2) | 1 (50.0)  (1.3, 98.7) | 0  (0.0, 70.8) | 1 (10.0) (0.3, 44.5) |
| DCR, n (%), (95%CI) | 1 (33.3)  (0.8, 90.6) | 0  (0.0, 84.2) | 1 (50.0)  (1.3, 98.7) | 0  (0.0, 70.8) | 2 (20.0) (2.5, 55.6) |
| Median PFS (95%CI), months | 2.1  (2.0, NA) | 2.0  (1.7, NA) | NA  (1.2, NA) | 1.7  (1.2, NA) | 2.1 (1.2, NA) |
| Median DoR (95%CI), months | NA  (NA, NA) | NA  (NA, NA) | NA  (NA, NA) | NA  (NA, NA) | NA (NA, NA) |

CR, complete response; PR, partial response; SD, stable disease; PD, progressive disease; NE, not evaluable; ORR, objective response rate; CI, confidence interval; DCR, disease control rate; PFS, progression-free survival; DoR, duration of response; NA, not available.


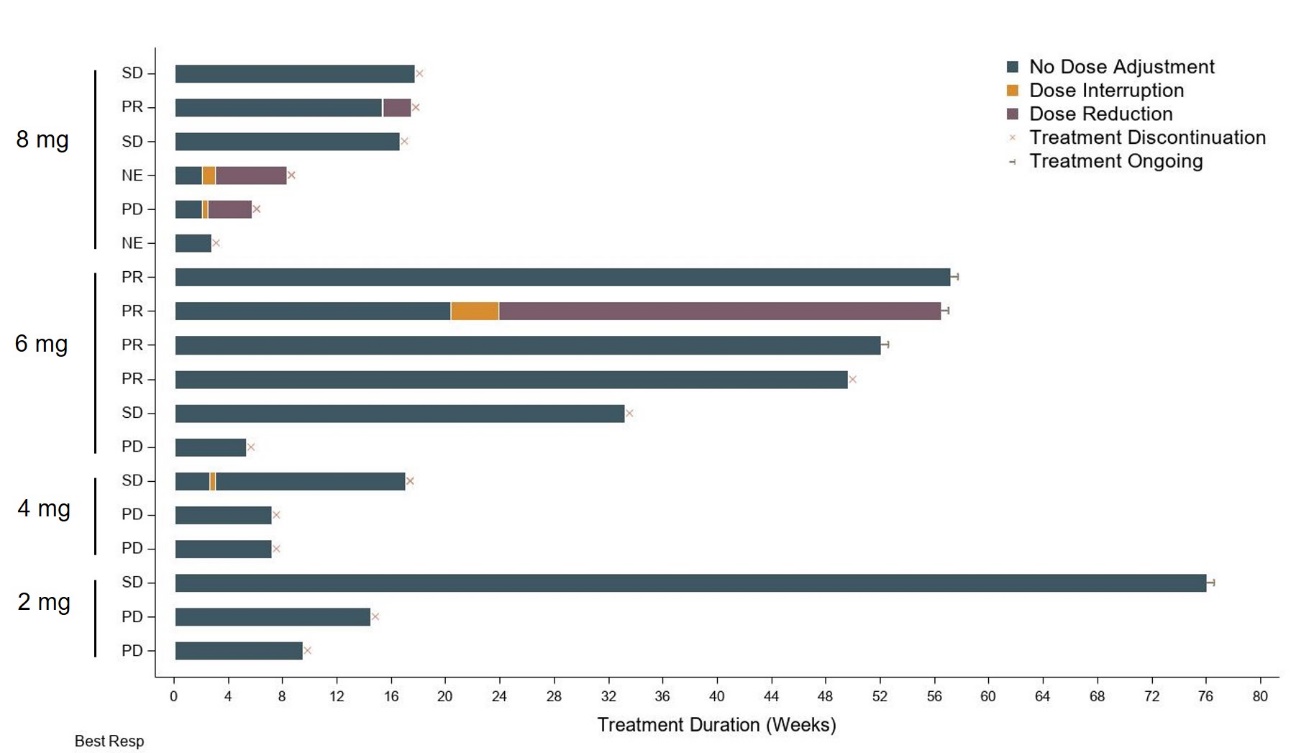


**Supplementary figure 1.** Swimmer plot for the duration of treatment in individual patients. SD, stable disease; PR, partial response; NE, not evaluable; PD, progressive disease.

**
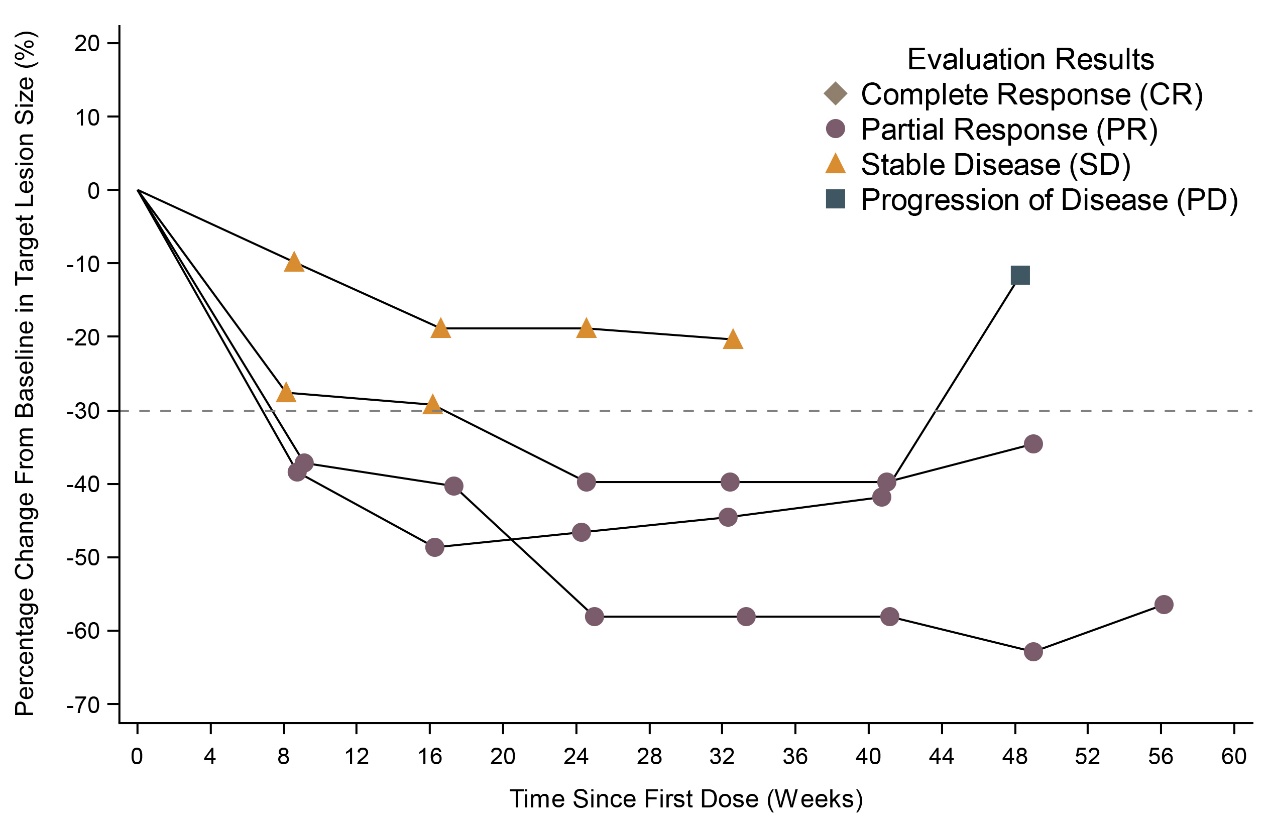
**

**Supplementary figure 2.** Spider plot for 4 patients (6 mg QD) with PIK3CA mutated tumors confirmed by the central laboratory.


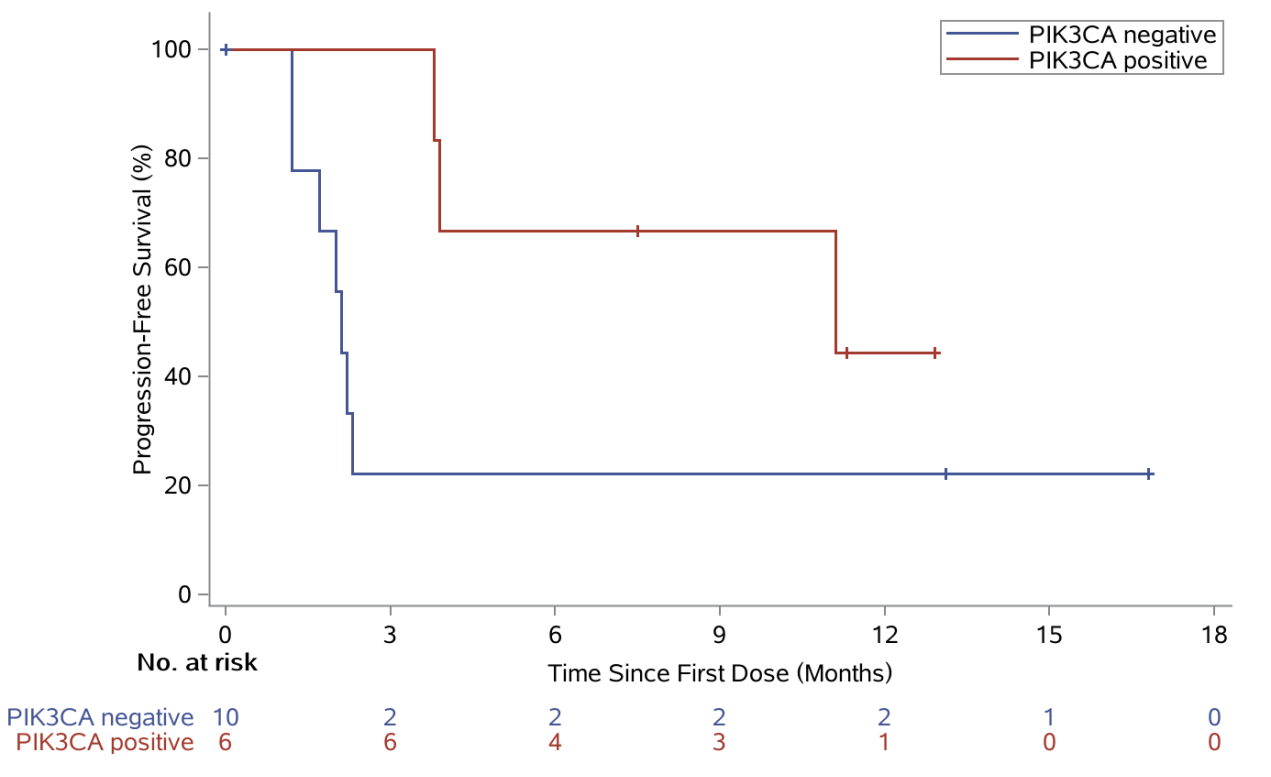


**Supplementary figure 3.** Progression-free survival in patients with PIK3CA-positive and PIK3CA-negative tumors


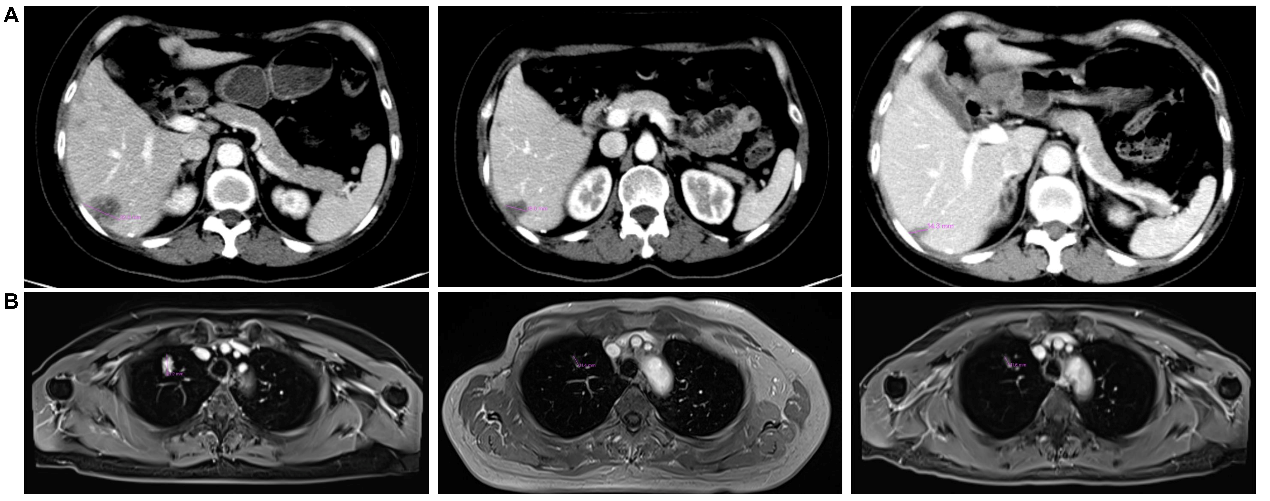


**Supplementary figure 4.** Imaging in patients with a partial response. (A) A patient with positive PIK3CA in the 6 mg QD group had a PR on Day 1 of Cycle 3 (C3D1), and the target lesion continued to shrink in subsequent follow-up visits, maintaining a PR on C15D1. (B) Another patient with positive PIK3CA in the 6 mg QD group had target lesion shrinkage on C3D1 and had a PR on C7D1, maintaining a PR on C13D1.
